# Human Blastocyst Components Detection Using Multiscale Aggregation Semantic Segmentation Network for Embryonic Analysis

**DOI:** 10.3390/biomedicines10071717

**Published:** 2022-07-15

**Authors:** Muhammad Arsalan, Adnan Haider, Se Woon Cho, Yu Hwan Kim, Kang Ryoung Park

**Affiliations:** Division of Electronics and Electrical Engineering, Dongguk University, 30 Pildong-ro, 1-gil, Jung-gu, Seoul 04620, Korea; arsal@dongguk.edu (M.A.); adnanhaider@dgu.ac.kr (A.H.); jsu319@naver.com (S.W.C.); taekkuon@naver.com (Y.H.K.)

**Keywords:** human blastocyst, infertility, embryo, semantic segmentation, in vitro fertilization

## Abstract

Infertility is one of the most important health concerns worldwide. It is characterized by not being successful of pregnancy after some periods of periodic unprotected sexual intercourse. In vitro fertilization (IVF) is an assisted reproduction technique that efficiently addresses infertility. IVF replaces the actual mode of reproduction through a manual procedure wherein embryos are cultivated in a controlled laboratory environment until they reach the blastocyst stage. The standard IVF procedure includes the transfer of one or two blastocysts from several blastocysts that are grown in a controlled environment. The morphometric properties of blastocysts with their compartments such as trophectoderm (TE), zona pellucida (ZP), inner cell mass (ICM), and blastocoel (BL), are analyzed through manual microscopic analysis to predict viability. Deep learning has been extensively used for medical diagnosis and analysis and can be a powerful tool to automate the morphological analysis of human blastocysts. However, the existing approaches are inaccurate and require extensive preprocessing and expensive architectures. Thus, to cope with the automatic detection of blastocyst components, this study proposed a novel multiscale aggregation semantic segmentation network (MASS-Net) that combined four different scales via depth-wise concatenation. The extensive use of depthwise separable convolutions resulted in a decrease in the number of trainable parameters. Further, the innovative multiscale design provided rich spatial information of different resolutions, thereby achieving good segmentation performance without a very deep architecture. MASS-Net utilized 2.06 million trainable parameters and accurately detects TE, ZP, ICM, and BL without using preprocessing stages. Moreover, it can provide a separate binary mask for each blastocyst component simultaneously, and these masks provide the structure of each component for embryonic analysis. Further, the proposed MASS-Net was evaluated using publicly available human blastocyst (microscopic) imaging data. The experimental results revealed that it can effectively detect TE, ZP, ICM, and BL with mean Jaccard indices of 79.08, 84.69, 85.88%, and 89.28%, respectively, for embryological analysis, which was higher than those of the state-of-the-art methods.

## 1. Introduction

Infertility is a medical health condition characterized by not being successful of pregnancy after a year of sufficient sexual intercourse without protection [1]. In China, the infertility prevalence was 16.4% and it is expected to increase up to 18.2% by 2023 [2]. Over the years, multiple schemes have been proposed to deal with infertility. These schemes are collectively referred to as assisted reproductive technologies (ART). In vitro fertilization (IVF) is considered the most effective ART that is commonly utilized to deal with infertility [3]. IVF is a manual reproductive technique in which embryos are cultivated outside the human body in a controlled laboratory environment until they reach the blastocyst stage. Then, these embryos are transferred back to the uterus of the patient [4]. The morphological attributes and formation of the specific embryo compartments are the indications of the embryo reaching the blastocyst stage. Based on these formations and developmental capabilities the transfer of viable embryos can increase the chance of pregnancy by IVF [5]. Conventionally, embryologists use time-lapse microscopic analysis to assess the specific composition of the blastocyst and the proper formation of its specific compartments. The formation of these compartments (such as trophectoderm (TE), zona pellucida (ZP), inner cell mass (ICM), and blastocoel (BL)) is a sign of implantation and development capabilities of a blastocyst.

TE is the external layer of a mammalian blastocyst that provides the nutrients for embryo development, and it protects the ICM from the outer environment. Studies have shown that morphological properties and scores can be correlated with clinical implantation rate in IVF [6]. ZP contains an extracellular glycoprotein matrix that manages sperm-egg interaction and encapsulates the oocyte, including ZP1, ZP2, ZP3, and ZP4 [7]. Further, ZP morphology is important for selecting viable blastocysts [8]. ICM contains the pluripotent epiblast (EPI), which is covered by a thin layer of endoderm. This endoderm, EPI, and TE give rise to the embryo to form a yolk sac and placenta before being transferred to the uterus [9]. In addition, ICM formation and morphological development provide effective evidence for viability testing of an embryo [10]. BL, an important component of the blastocyst, is a fluid cavity created on the fifth day. Its creation indicates that the embryo has converted to the blastocyst stage and, at this stage, the ICM is situated on the side of the blastocyst. Moreover, BL morphometric properties are important for the embryological analysis of IVF [11]. The embryologist finally evaluates the viability of the blastocyst based on these aforementioned properties [12]. According to blastocyst euploid selective transfer (BEST) trials, the success rate of single euploid blastocyst transfer was 69% and 61% for clinical pregnancy and ongoing pregnancy, respectively, whereas the success rate of untested 2-blastocyst transfer was 81%, and 65% for clinical pregnancy and ongoing pregnancy, respectively [13,14]. A single-embryo transfer is considered safe as it helps to avoid maternofetal risks [15]. Blastocyst competence assessment is important to determine the best single embryo that has the highest potential for pregnancy [16].

The manual embryonic analysis is time-consuming and requires continuous keen observations and subject knowledge. In the current era of machine learning and artificial intelligence (AI), deep-learning-based methods aid humans with several medical applications [17]. Thus, AI can help in the assessment of sperm, embryos, and oocytes to improve the success rate of IVF [18]. Deep-learning-based semantic segmentation can help pixel-wise detection of blastocyst compartments (TE, ZP, ICM, and BL) for morphological analysis. Very few methods have been developed based on semantic segmentation, with most involving expensive architectures for detecting these compartments. To address these issues, this study proposed a multiscale aggregation network (MASS-Net) that combined four different scales for valuable spatial information aggregation to accurately detect TE, ZP, ICM, and BL in blastocyst images. MASS-Net is based on a few layers (a combination of 33 general convolutions and depth-wise separable convolutions (DWSC)). MASS-Net includes the following important provisions:

- It detects the blastocyst components without using conventional image processing schemes.
- It is a multiscale semantic segmentation network that uses four different scales without increasing the depth of the network.
- The feature boost block (FBB) helps pick the boundaries of the components (TE, ZP, ICM, and BL) that are not easily discernible.
- The proposed MASS-Net trained semantic segmentation models were made publicly available in [19].

The remainder of this paper is organized as follows. Section 2 provides insights into materials and methods. Section 3 presents the results of the proposed MASS-Net. Section 4 presents the discussion. Finally, Section 5 presents the conclusions of the study.

## 2. Materials and Methods

### 2.1. Datasets

**Figure 1:**
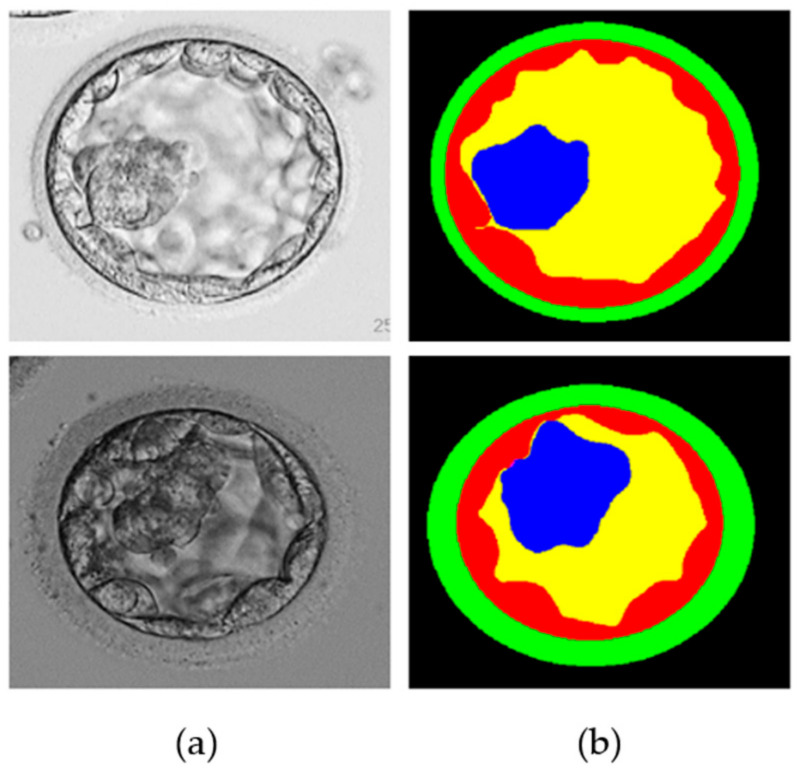
Example microscopic blastocyst image used in the experiments. (**a**) Original images; (**b**) expert annotations for blastocyst components of TE (shown by red color), ZP (shown by green color), ICM (shown by blue color), BL (shown by yellow color), and background (shown by black color).

To verify the performance of the proposed MASS-Net, a publicly available blastocyst microscopic image dataset introduced in [20] was used. This dataset included human blastocyst images with pixel-level annotations of TE, ZP, ICM, and BL acquired by an expert embryologist. Moreover, these images and multiclass annotations are publicly available for research purposes [20]. The dataset comprises 235 Hoffman modulation contrast microscopic images at magnifications of 1.6×, and 20× objective lenses. These images were captured for different patients between 2012 and 2016 using an Olympus IX71 (Olympus Corp., Tokyo, Japan) inverted microscope and Research Instrument Cronus 4 software (Research Instruments, Falmouth, England) at the Pacific Center for Reproduction Canada. In addition, to realize a fair comparison with the state-of-the-art learning-based method on the same dataset, the train-test split method mentioned in [21,22] was enforced. Out of a total of 235 images, 200 (85%) and 35 (15%) were used for training and testing, respectively. Figure 1 shows an example image and an expert annotation image. However, arranging many training examples in terms of medical images to sufficiently train a semantic segmentation network is challenging. Therefore, image augmentation was used to create synthetic images using various image operations (flipping, rotations, and image translations) to create 3200 images from 200 training images.

### 2.2. Overview of the Proposed MASS-Net-Based Segmentation of Blastocyst Components

**Figure 2:**
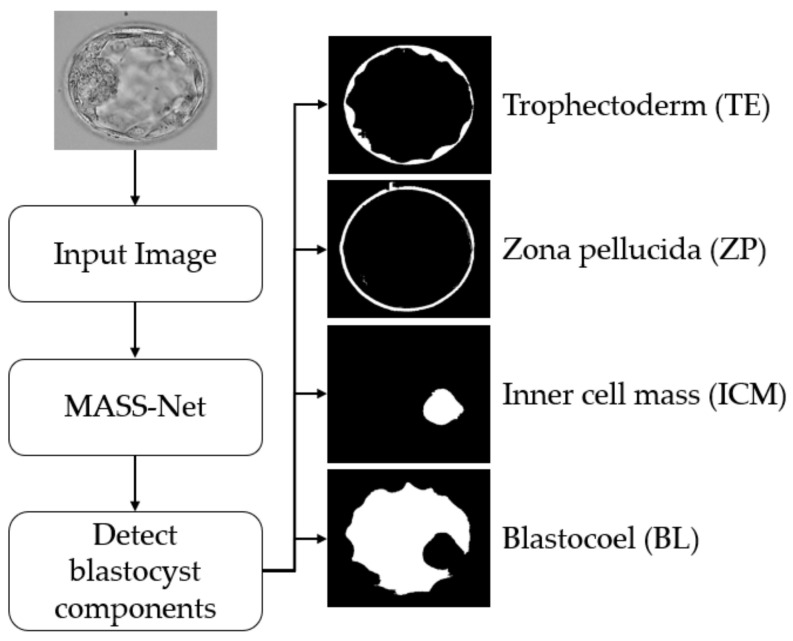
Summarized workflow of the proposed method (MASS-Net) for embryo component segmentation for embryonic analysis. Abbreviation: MASS-Net, multiscale aggregation semantic segmentation network.

This study proposed a deep-learning-based semantic segmentation method for blastocyst component detection that can be used for embryological analysis through morphometric properties. Figure 2 shows the overall workflow of the proposed MASS-Net-based embryo component detection, which is based on multiscale aggregation using the depth-wise (channel-wise) concatenation of features. The multiscale aggregation provides different resolution scales of spatial information combined to create rich features for detecting embryo components without preprocessing. Microscopic embryo images are of inferior quality and these components are not easily discernible. However, the effective design of MASS-Net with rich spatial information learning aids the network in identifying these pixels. According to Figure 2, MASS-Net acquires the blastocyst image at the input without image enhancement and applies the multiscale convolutional operation. Thereafter, at the output, the network provides four individual masks for the TE, ZP, ICM, and BL. The binary mask for each component assigns the positive class as ‘1’ and other pixels as ‘0’. These masks represent the morphology of a specific component that can be used to assess morphometric properties for viability checks.

### 2.3. MASS-Net Design Principles

In general, semantic segmentation is performed with an encoder-decoder structure wherein the decoder is the same as the decoder. Certain examples of encoder-decoder structures are SegNet [23] and U-Net [24]. However, as these conventional networks are designed for various general tasks, they cannot perform well for a specific task where minor classes, such as ZP or ICM, are available. Moreover, conventional semantic segmentation design is based on very deep networks (using many convolutional layers) that consume many trainable parameters [23,24,25]. Consequently, networks that are sufficiently deep and utilize many convolutional layers are prone to spatial information loss. In addition, intensive use of pooling layers causes spatial size reduction that can remove minor features during training.

**Figure 3:**
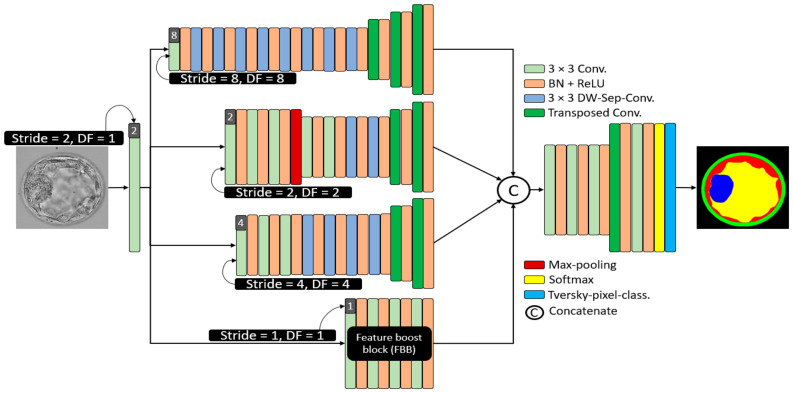
The architecture of the proposed MASS-Net. Here in this figure 3 × 3 kernel convolution (3 × 3 conv.), batch normalization in combination with rectified linear unit (BN + ReLU), 2 × 2 transposed convolutions with stride = 2 (Transposed Conv.), and 3 × 3 kernel-based DWSC (DW-Sep-Conv.).

Considering embryo component segmentation, these components have very close gray levels and indistinctive boundaries. Moreover, these components can be excessively small, and thus, with feature empowerment, they cannot be effectively detected via networks that use multiple pooling layers. The structure of MASS-Net is shown in Figure 3 to explain the connectivity of the downsampling and upsampling blocks. MASS-Net considers four main design principles for effective semantic segmentation architecture. First, the semantic segmentation model, which is based on a low number of trainable parameters. MASS-Net extensively uses DWSC with a shallow decoder that helps reduce the number of trainable parameters. Second, the extensive use of pooling layers causes spatial information loss [26], which prevents the extensive usage of pooling layers and uses strided convolutions with learned weights for better performance. Third, multiscale architectures can extract valuable context information using different image resolutions, and a combination of these scales can improve segmentation performance [27,28]. MASS-Net has a low number of trainable parameters and a network that may suffer segmentation performance on a single scale. Thus, the multiscale aggregation (Scale-8, Scale-4, Scale-4, Scale-2, Scale-1) provides a rich combined feature of these scales to boost the segmentation performance. Fourth, the components (ZP, TE, ICM) can be very small and can be partially or completely eliminated. MASS-Net uses a feature booster block (FBB) that uses few convolutional layers without reducing the spatial dimension of the feature, which helps retain these small components in the image.

### 2.4. Structure of Proposed MASS-Net Downsampling Block

The Scale-8 (shown by the top block of Figure 3) was created by a 3 × 3 convolution with stride value and dilation factor of 8 each (S = 8, DF = 8). The Scale-8 block comprised eight low-cost DWSC and three transposed convolutions for upsampling three times. Further, the Scale-2 (shown by the second block from the top of Figure 3) was created by a 3 × 3 convolution with stride value and dilation factor of 2 each (S = 2, DF = 2). The Scale-2 block comprised five 3 × 3 convolutions, two DWSC, two transposed convolutions for upsampling, and one max-pooling layer. Furthermore, the Scale-4 (shown by the third block from the top of Figure 3) was created by a 3 × 3 convolution with stride value and dilation factor of 4 each (S = 4, DF = 4). The Scale-4 block comprised three further 3 × 3 convolutions, four DWSC, and two transposed convolutions for upsampling. In addition, the feature booster block (FBB) is a special block that retains the feature map size and uses a few convolutions for minor information retention. It uses four 3 × 3 convolutions with a few channels.

**Figure 4:**
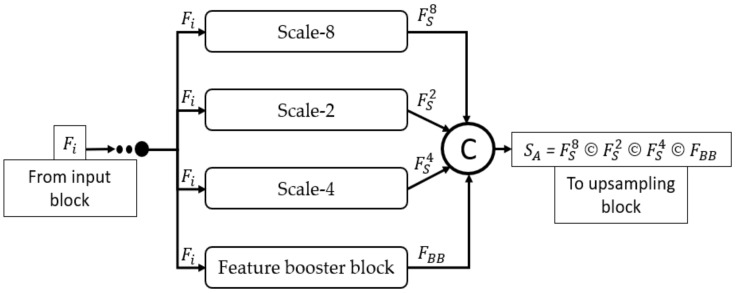
Dense connectivity pattern used in MASS-Net architecture for multiscale aggregation.

Figure 4 mathematically illustrates the four-scale connectivity pattern (Scale-8, Scale-2, Scale-4, and Scale-1 by FBB). The input block accepts the input image and outputs the $F_{i}$, a feature that is equally provided to each scale block, where each scale block outputs $F_{S}^{8}$, $F_{S}^{2},$ $F_{S}^{4}$, and $F_{BB}$ features for Scale-8, Scale-2, Scale-4, and FBB, respectively. These four features include multiscale spatial information that are combined to create a dense aggregated feature $S_{A}$ expressed as Equation (1).

(1) $$S_{A}\;=\;F_{S}^{8}\;©\;F_{S}^{2}\;©\;F_{S}^{4}\;©\;F_{BB}$$

where $S_{A}$ is the dense concatenated feature generated by the depth-wise (channel-wise) concatenation of $F_{S}^{8}$ (feature from Scale-8 block), $F_{S}^{2}$ (feature from Scale-2 block), $F_{S}^{4}$ (feature from Scale-4 block), and $F_{BB}$ (feature from feature booster block). Further, © represents the depthwise concatenation of these features. Considering the design principles of MASS-Net mentioned in Section 2.3, the proposed network used multiple DWSC to lower the trainable parameters. Consequently, the proposed MASS-Net consumes only 2.06 million trainable parameters, which is much lower than those of conventional networks.

Table 1 presents the information regarding feature map sizes for each block (layer-wise) to explain the different features of the proposed network. MASS-Net avoids multiple pooling operations, as shown in Figure 3, using only one max-pooling layer in the Scale-2 block. Further, it employs an FBB that is specifically designed to deal with minor information features. Moreover, according to Equation (1), the downsampling block provides a dense aggregated feature $S_{A}$ to the upsampling block.

### 2.5. Feature Booster Block (FBB)

Conventional networks use multiple pooling layers when diving deeper into the network. This pooling phenomenon can benefit larger classes that are available in an image. The SegNet [23], uses VGG16 as the backbone and five pooling layers. However, although SegNet performs extremely well for larger classes available in the image (building, sky, road, etc.), this is not true for the minor classes available in the image (column pole, bicyclist, etc.). This is primarily due to the extensive usage of pooling layers and the unavailability of feature empowerment. Thus, the FBB in MASS-Net specifically addresses the feature degradation issue for the minor classes available in blastocyst microscopic images. It is evident from Table 1 that FBB maintains the feature map size at 200 × 200. This feature map size is sufficient to represent minor features (ICM, TE, and ZP) in the image. Therefore, FBB empowerment boosts segmentation performance.

### 2.6. Structure of MASS-Net Upsampling Block

The main objective of MASS-Net was to achieve high segmentation performance for reliable embryological analysis in addition to designing a network that consumes a low number of trainable parameters. The downsampling block already considers different scales whereas the FBB block boosts the feature that helps retain the minor classes. Moreover, MASS-Net consists of very few convolutional layers and just one transposed convolution. This shallow upsampling block help to lower the trainable parameters. It is evident from Table 1 that the upsampling block contains the final convolution with five filters that represent the number of classes (TE, ZP, ICM, BL, and background) that are considered by the network. The MASS-Net provides each class mask with these five filters. As shown in Figure 2 there is a large pixel difference between all of the classes, with the background and BL classes having many pixels compared to ZP, TE, and ICM. This pixel difference creates the class imbalance, which can be addressed by using an appropriate loss function. This study utilized the Tversky loss function [29], which can handle the class imbalance effectively. The details for Tversky loss are provided in [29].

### 2.7. Training of Proposed Method, Experimental Environment and Protocols

**Figure 5:**
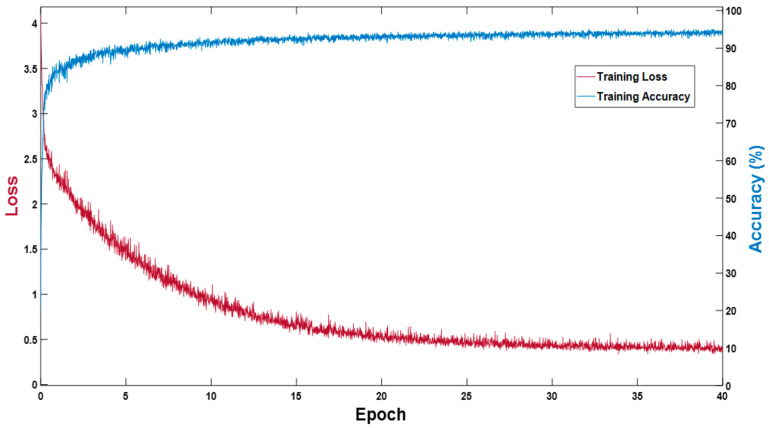
MASS-Net training accuracy-loss curve.

The proposed MASS-Net does not use weight migration or fine-tuning; rather, it is trained from scratch. In the experiments performed, Adam [30] was used as an optimizer to train 3600 (40 epochs) with an initial learning rate (ILR) of 0.0001, a mini-batch size of 20 images with an epsilon of 0.000001, and global L2-normalization. In the training experiments, image shuffling was used to provide variations for each epoch. Figure 5 presents the training accuracy-loss curve for MASS-Net, which shows that MASS-Net attained a high training accuracy with lower training loss.

### 2.8. Evaluation of Proposed Method (MASS-Net)

As shown in Table 1 Class-Mask-Conv layers have five filters; therefore, MASS-Net produced five masks at the output of the network for the ICM, TE, ZP, BL, and background. These masks represent the blastocyst component pixels by “1” and all of the background pixels with “0” for each class. Subsequently, to evaluate the segmentation performance, the output masks of MASS-Net were pixel-wise compared with expert annotation (provided by an expert embryologist), and a versatile Jaccard index (JI) was used to compute the performance. MASS-Net is a learning-based method; therefore, following [21,22], JI was used to fairly compare the proposed method with state-of-the-art methods on the same dataset and training-testing protocols. The JI is expressed Equation (2): where true-positive (TP) is a pixel that is predicted as an embryo component pixel and embryo pixel in the expert annotation. Whereas, a false positive (FP) is a pixel that is predicted as an embryo component pixel, and is not an embryo pixel in the expert annotation. Finally, a false negative (FN) is a pixel that is predicted as a non-embryo pixel, and it is an embryo component pixel in the expert annotation. (# show the number pixels)

(2) $$\mathrm{Jaccard}\;\mathrm{Index}\;\left(\mathrm{JI}\right)\;=\;\frac{\;\#\mathrm{TP}}{\#\left(\mathrm{TP}\;+\;\mathrm{FP}\;+\;\mathrm{FN}\right)}$$

## 3. Results

### 3.1. Ablation Study for MASS-Net

Two types of ablations were conducted to prove the efficacy of the proposed MASS-Net. Multiple uses of image size reduction by pooling layers or strided convolutions can eliminate minor information from the image. The FBB (explained in Section 2.5) retains a feature map size of 200 × 200 pixels, which is sufficient for minor features in the image. To prove the effectiveness of the FBB, the first ablation experiment was conducted with and without the FBB. It is evident from Table 2 that the FBB improved the overall segmentation performance while consuming a few trainable parameters. The segmentation performance of fully convolutional networks decorates with the class imbalance in the dataset. Several schemes can be used to deal with class imbalance, such as weighted cross-entropy (WCE) [31], focal loss (FL) [32], dice loss (DL) [33], and TVL [29]. In the second type of ablation, different loss functions were tested for embryo component segmentation using the MASS-Net. It is evident from Table 2 that MASS-Net with TVL provided the best results while using only 2.06 million trainable parameters overall.

### 3.2. Comparison of MASS-Net with State of the Art Methods

This section provides a comparison of the proposed MASS-Net with state-of-the-art methods for human blastocyst component detection. Table 3 presents the numerical performance assessment and comparison of the microscopic blastocyst images. Table 3 is based on JI described in Section 2.8.

### 3.3. Visual Results of Proposed MASS-Net for Embryonic Component Segmentation

**Figure 6:**
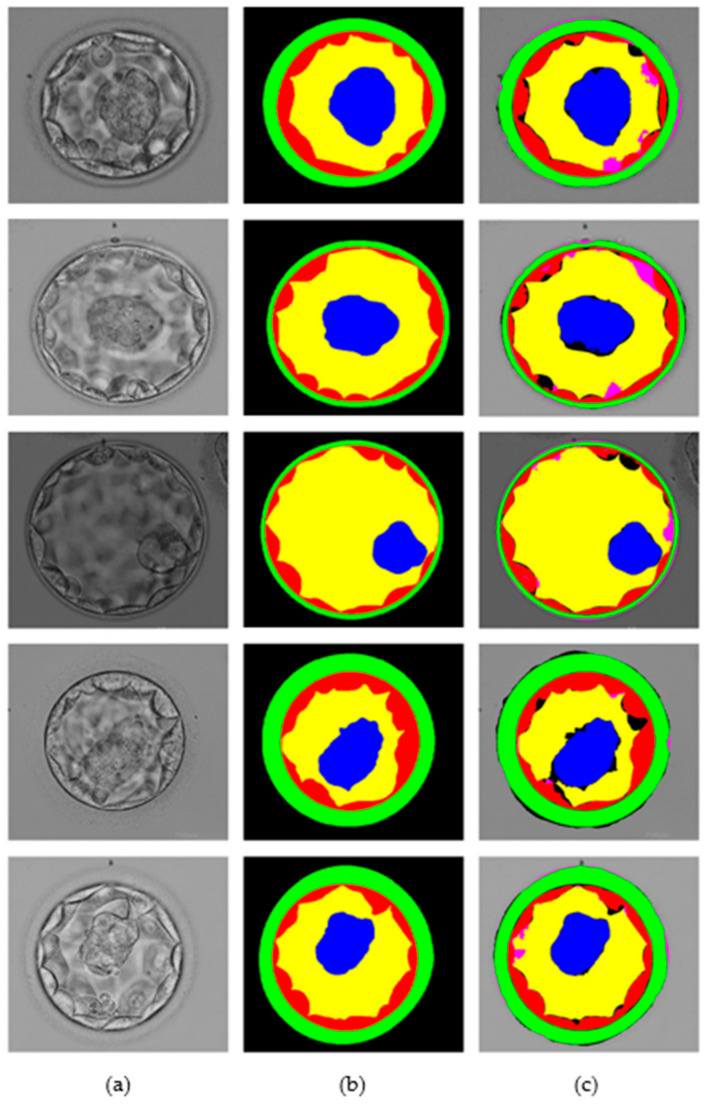
MASS-Net visual results for embryo component segmentation: (**a**) input microscopic blastocyst image, (**b**) expert annotation, and (**c**) MASS-Net multiclass predicted mask.

This section presents the blastocyst segmentation visual results provided by the proposed MASS-Net on a publicly available microscopic blastocyst image dataset. Figure 6 presents MASS-Net segmentation visual results in comparison with expert annotation; where (a) input blastocyst image, (b) medical expert annotation for TE, ZP, ICM, BL, and BG, and (c) MASS-Net multiclass predicted masks are displayed.

## 4. Discussion

The semantic segmentation is a specific machine learning procedure that deals with pixel-wise classification. Dealing with the segmentation for minor classes is challenging, and conventionally the depth of the network is increased to get good segmentation performance. The number of trainable parameters substantially increases as we increase the depth of the network (using more layers). It can be noticed from Table 3 the proposed MASS-Net is using only 2.06 million trainable parameters, and these parameters are much lower than the famous semantic segmentation methods presented in Table 3. The proposed MASS-Net is a step towards development of mobile low-cost platform. However, it is very difficult to manage good segmentation performance with low number of parameters. MASS-Net with effective multiscale design and feature booster block manages the competitive segmentation performance with reduced depth of the network.

### 4.1. Visual Representation of Predictions

**Figure 7:**
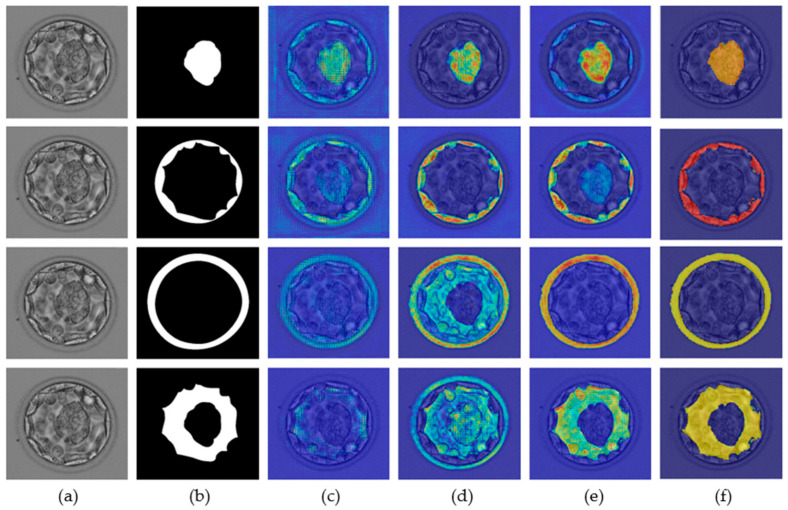
MASS-Net Grad-CAM visualization for ICM (row-1), TE (row-2), ZP (row-3), and BL (row-4): (**a**) original blastocyst microscopic image, (**b**) expert annotation, and Grad-CAM obtained from the ReLU of (**c**) S2-Tconv-B, (**d**) US-Conv-A, (**e**) US-Conv-B, and (**f**) US-Tconv-A.

A neural network predicts a label based on a specific feature for a specific class. Recognition of these specific features is extremely important for the successful development of custom networks. The ions from the network are based on specific features, and these features are important for the development of a robust segmentation network. The gradient-weighted class activation map (Grad-CAM) [36] helps highlight the features that contribute to the prediction of a label. The neural network gradually learns the related features, and as one enters the deeper layers, this learning improves. Figure 7 presents the Grad-CAM blastocyst images. These Grad-CAMs were extracted from the rectified linear units (ReLUs) of the four different layers of Table 1 (S2-Tconv-B, US-Conv-A, US-Conv-B, and US-Tconv-A). Grad-CAM shows that the proposed MASS-Net gradually learns the pixels of all blastocyst components without bias.

### 4.2. Embryonic Analysis

**Figure 8:**
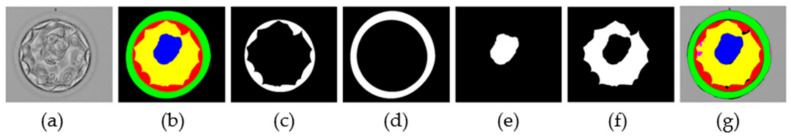
MASS-Net individual masks: (**a**) original image, (**b**) expert annotation by embryologist, (**c**) TE predicted mask, (**d**) ZP predicted mask, (**e**) ICM predicted mask, (**f**) BL predicted mask, and (**g**) combined predicted mask.

The morphological properties of blastocyst components are extremely important for determining embryo viability [6,21,37]. The MASS-Net effectively detects these components in a multiclass scenario. MASS-Net outputs individual binary masks for each component. Figure 8 presents examples of the output masks produced by MASS-Net. In these masks, each embryo component is represented by ‘1′ (white pixels), and the non-embryo component ‘0’ (black pixels). These masks provide accurate pixel-wise detection that can be used to analyze morphometric properties that are important for testing embryo viability. Moreover, the creation of a specific compartment can be detected automatically. Thus, an embryologist can analyze these MASS-Net-provided masks that can aid in collective assessment before transferring the embryo to the patient’s uterus.

## 5. Conclusions

The primary objective of this study was to present a novel and effective network for detecting blastocyst components using a simpler multiclass network. MASS-Net is a multiclass network that utilized different scales to aggregate them to render a powerful feature through dense concatenation. The FBB was based on a few convolutions, thus retaining a larger spatial size. The FBB provided rich low-level feature information, and this spatial information was added to the other scales, resulting in enhanced segmentation performance. Further, the intensive use of depth-wise separable convolutions and shallow upsampling blocks helped reduce the overall number of trainable parameters. Collectively, MASS-Net provided an accurate segmentation of TE, ZP, ICM, and BL for embryonic viability assessment.

The proposed method can extract the blastocyst components exactly from the embryo images, and these results are pretty close to medical expert annotation. Currently, this system cannot directly provide the quality (scoring) of the blastocyst, but it can help the embryologist in decision making. For example, the proposed method can detect if that component is formed (available). Our proposed method can directly predict the blastocyst quality if the training data is provided with blastocyst quality annotation. We are using a publicly available dataset (without blastocyst quality score annotation). Therefore, the current method can be used to aid the embryologist.

In the future, we have a plan to directly collaborate with the medical institutions to collect the data with blastocyst quality score annotations. Furthermore, our proposed method is a step toward the development of a mobile low-cost system. A similar system can be used to predict embryo quality using a shallow cost-effective architecture. In addition, this multiscale network will be utilized for the segmentation and analysis of other medical diseases. Furthermore, similar feature booster-based methods will be developed to further reduce the number of trainable parameters to create a low-cost mobile system for embryonic analysis.

## Figures and Tables

**Table 1:** Details of feature map sizes for MASS-Net architecture.

Block	Layer	Size of Layer K × K × C, (Stride)	Filters/Groups	Repetition	Output Size
	Input Conv	3 × 3 × 1 (S = 2)	16	1	200 × 200 × 16
Scale-8 block	S8-Conv-S	3 × 3 × 16 (S = 8)	32	1	25 × 25 × 32
	S8-DWSC	3 × 3 × 32 (S = 1)	32	8	25 × 25 × 32
	S8-Tconv-A	2 × 2 × 32 (S = 2)	32	1	50 × 50 × 32
	S8-Tconv-B	2 × 2 × 32 (S = 2)	32	1	100 × 100 × 32
	S8-Tconv-C	2 × 2 × 32 (S = 2)	32	1	200 × 200 × 32
Scale-2 block	S2-Conv-S	3 × 3 × 16 (S = 8)	32	1	100 × 100 × 32
	S2-Conv-A	3 × 3 × 32 (S = 1)	64	1	100 × 100 × 64
	S2-Conv-B	3 × 3 × 64 (S = 1)	64	1	100 × 100 × 64
	Pool	2 × 2 × 64 (S = 2)	-	1	50 × 50 × 64
	S2-Conv-C	3 × 3 × 64 (S = 1)	128	1	50 × 50 × 128
	S2-Conv-D	3 × 3 × 128 (S = 1)	256	1	50 × 50 × 256
	S2-DWSC	3 × 3 × 256 (S = 1)	256	2	50 × 50 × 256
	S2-Tconv-A	2 × 2 × 256 (S = 2)	128	1	100 × 100 × 128
	S2-Tconv-B	2 × 2 × 128 (S = 2)	64	1	200 × 200 × 64
Scale-4 block	S4-Conv-S	3 × 3 × 16 (S = 4)	32	1	50 × 50 × 32
	S4-Conv-A	3 × 3 × 32 (S = 1)	64	1	50 × 50 × 64
	S4-Conv-B	3 × 3 × 64 (S = 1)	128	1	50 × 50 × 128
	S4-DWSC-A	3 × 3 × 128 (S = 1)	128	2	50 × 50 × 128
	S4-DWSC-B	3 × 3 × 128 (S = 1)	64	2	50 × 50 × 64
	S4-Tconv-A	2 × 2 × 64 (S = 2)	128	1	100 × 100 × 128
	S4-Tconv-B	2 × 2 × 128 (S = 2)	64	1	200 × 200 × 64
Feature booster block	FBB-Conv-A	3 × 3 × 16 (S = 1)	32	1	200 × 200 × 32
	FBB-Conv-B	3 × 3 × 32 (S = 1)	64	1	200 × 200 × 64
	FBB-Conv-C	3 × 3 × 64 (S = 1)	64	1	200 × 200 × 64
	FBB-Conv-D	3 × 3 × 64 (S = 1)	128	1	200 × 200 × 128
Feature Aggregation	S8-Tconv-C © S2-Tconv-B © S4-Tconv-B © FBB-Conv-D				200 × 200 × 288
Upsampling block	US-Conv-A	3 × 3 × 288 (S = 1)	256	1	200 × 200 × 256
	US-Conv-B	3 × 3 × 256 (S = 1)	128	1	200 × 200 × 128
	US-Conv-C	3 × 3 × 128 (S = 1)	64	1	200 × 200 × 64
	US-Tconv-A	2 × 2 × 64 (S = 2)	32	1	400 × 400 × 32
Final masks	Class-Mask-Conv	1 × 1 × 32 (S = 1)	5	1	400 × 400 × 5

**Table 2:** MASS-Net ablation study results. (#Pram. show the number of trainable parameters).

Method	TE	ZP	ICM	BL	BG	Mean JI	#Pram.
MASS-Net (WCE)	77.34	82.14	85.13	87.98	95.86	85.69	2.06 M
MASS-Net (FL)	76.88	85.09	83.70	86.60	90.97	84.65	2.06 M
MASS-Net (DL)	78.98	84.12	84.68	88.92	95.61	86.46	2.06 M
MASS-Net (TVL without FBB)	77.25	84.76	84.55	87.78	95.96	86.06	1.63 M
MASS-Net (TVL with FBB)	79.08	84.69	85.88	89.28	96.07	87.00	2.06 M

**Table 3:** Performance comparison of proposed MASS-Net with current state-of-the-art methods for blastocyst component segmentation. (#Pram. show the number of trainable parameters).

Method	TE	ZP	ICM	BL	BG	Mean JI	#Pram.
U-Net (baseline) [24]	75.06	79.32	79.03	79.41	94.04	81.37	31.03 M
Ternaus U-Net [34]	76.16	80.24	77.58	78.61	94.50	81.42	10 M
PSP-Net [35]	74.83	80.57	78.28	79.26	94.60	81.51	35 M
DeepLab-V3 [25]	73.98	80.84	80.60	78.35	94.49	81.65	40 M
BlastNet [22]	76.52	81.15	81.07	80.79	94.74	82.85	25 M
SSS-Net (Residual) [21]	77.40	82.88	84.94	88.39	96.03	85.93	4.04 M
SSS-Net (Dense) [21]	78.15	84.51	84.50	88.68	95.82	86.34	4.04 M
MASS-Net (Proposed without FBB)	77.25	84.76	84.55	87.78	95.96	86.06	1.63 M
MASS-Net (Proposed with FBB)	79.08	84.69	85.88	89.28	96.07	87.00	2.06 M

## Data Availability

Not applicable.
